# Pro-apoptotic effect of doxycycline and hydroxychloroquine on B-cell lymphoma induced by *C. burnetii*

**DOI:** 10.18632/oncotarget.23397

**Published:** 2017-12-18

**Authors:** Cléa Melenotte, Didier Raoult

**Affiliations:** ^1^ Aix Marseille Université, CNRS, IRD, INSERM, AP-HM, Unité de Recherche sur les Maladies Infectieuses et Tropicales Emergentes, Institut Hospitalier Universitaire Méditerranée-Infection, Marseille, France

**Keywords:** apoptosis, non hodgkin lymphoma, Q fever, doxycycline, hydroxychloroquine

The combination of doxycycline with hydroxychloroquine is the cornerstone of treatment for persistent *Coxiella burnetii* infection, and we have used this combination for years in the National Reference Center for Q fever. While *C. burnetii* blocks apoptosis of the host cells in order to survive and proliferate, doxycycline on the other hand has a pro-apoptotic effect [1, 2].

**Figure 1:**
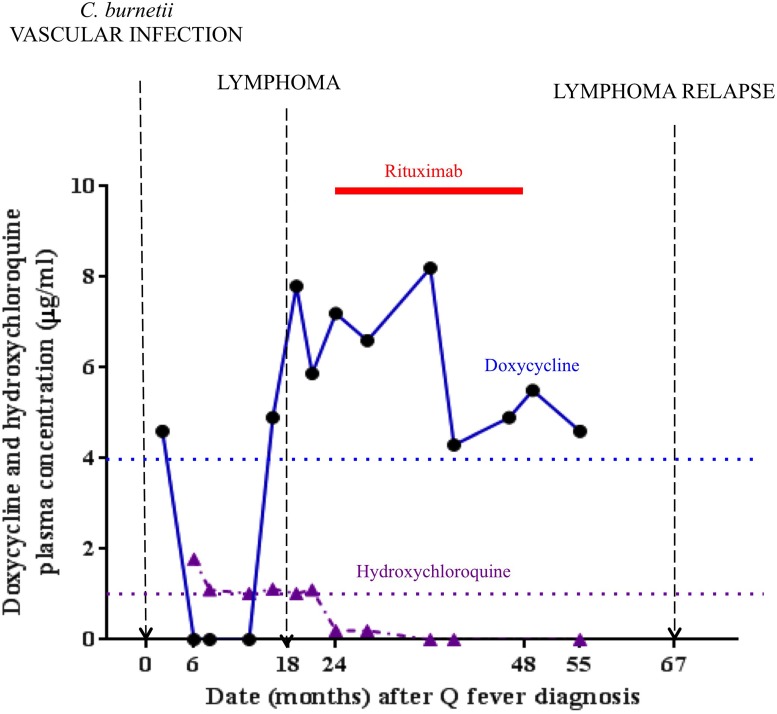
Doxycycline and hydroxychloroquine serum levels concentrations in the index patient The therapeutic threshold is defined by the dotted bar as 4 μg/ml for doxycycline and 1 μg/ml for hydroxychloroquine.

We recently described the history of a patient with a *C. burnetii* vascular infection who 18 months later developed B-cell non-Hodgkin lymphoma [3]. At the date of the Q fever diagnosis, doxycycline 200 mg once per day in combination with hydroxychloroquine 200 mg three times per day was introduced. Analyzing antibiotic blood levels, we noted that plasma doxycycline levels were not detectable during the first year of the infection, while plasma hydroxychloroquine levels were within expected therapeutic values (Figure 1). Eighteen months after the diagnosis of *C. burnetii* vascular infection, follicular lymphoma was diagnosed on a retroperitoneal biopsy (Figure 1). Rituximab chemotherapy was instituted for two years, doxycycline was reintroduced and hydroxychloroquine was stopped. When rituximab was stopped, 4 years after the diagnosis of Q fever, the patient was in remission, and doxycycline was prolonged for a 7-month period (Figure 1). The patient was considered cured of the *C. burnetii* infection (phase I *C. burnetii* antibodies were 100, versus 800 at the time of the Q fever diagnosis), but the lymphoma recurred one year after doxycycline was stopped. Lymphoma developed while doxycycline was not effective and relapse was observed while doxycycline was stopped (Figure 1). This observation emphasizes the benefits of doxycycline as a pro-apoptotic medication.

To survive within host cells, *C. burnetii* utilizes an anti-apoptotic strategy. It establishes a replicative niche in a lysozyme-like parasitophorous acid vacuole that is permissive for bacterial replication [1]. At the same time, *C. burnetii* inhibits host cell apoptosis by reducing the levels of caspase-3. *C. burnetii*-infected cells have been associated with a decrease in pro-apoptotic bcl-2 family proteins such as Bax and Bak and a dramatically increased synthesis of the anti-apoptotic bcl-2 family protein A1/bfl1, which promotes mitochondrial outer membrane integrity [4]. Therefore, mitochondrial fission is inhibited, and levels of caspase-3, otherwise called “executioner” caspase, are reduced and apoptosis is thus inhibited [4].

On the other hand, doxycycline presents pro-apoptotic properties in addition to its antibacterial activity. In fact, doxycycline inhibits cell growth and promotes apoptosis in tumor cells by activating caspase-3 and caspase-9. Doxycycline is able to enhance the therapeutic activity of anticancer therapy, reduce its side effects, and prevent tumor formation [2, 5]. The anti-tumoral effect of doxycycline has been demonstrated in breast, ovarian, prostate, lung, pancreatic and colon cancer cells, and in melanoma, oral squamous cell carcinoma and in two-thirds of MALT lymphoma associated with *Chlamydia psittaci* [5]. Pulvino et al. have recently reported that doxycycline inhibits the growth of B-cell lymphoma in vitro and in vivo (in mice) for diffuse large B-cell lymphoma (DLBCL) and in vitro for several other types of non-Hodgkin lymphoma (NHL)(2). Doxycycline accumulates in DLBCL cells in high concentrations and affects crucial signaling pathways for lymphomagenesis; NF-kB, STAT3, ERK and the COP-9 signalosome CSN5, which are essential for DLBCL cell survival [2, 5].

Hydroxychloroquine potentiates the bactericidal activity of doxycycline by alkalinizing the vacuolar pH [4]. This antimalarial drug is found to be effective against bacterial and viral infections, but also has activity as an anti-inflammatory drug currently used in rheumatoid arthritis and systemic lupus erythematous. Antineoplastic effects of hydroxychloroquine have been demonstrated in reducing the incidence of Burkitt lymphoma in Tanzania, in preventing the development of B-cell lymphoma in mice and in inducing apoptosis of malignant B-cells in patients with chronic lymphocytic leukemia. Moreover, these antineoplastic effects have also been demonstrated in solid tumors such as breast and colorectal cancer [6] . Similar to doxycycline, hydroxychloroquine pro-apoptotic mechanisms result from caspase-3 activation and from bcl-2/bax ratio modulation [6].

Other intracellular pathogens are implicated in carcinogenesis: *M. tuberculosis*, *Chlamydia pneumoniae* and *Chlamydia psittaci* [4] . While doxycycline is currently used for *Chlamydia* infection, this antibiotic has no place in the treatment of *M. tuberculosis* infection. Like *C. burnetii*, *M. tuberculosis* down-regulates the anti-apoptotic bcl-2 protein in macrophages in order to survive and ensure sufficient bacterial replication [7]. The place of doxycycline and hydroxychloroquine in the treatment of *M. tuberculosis* infection deserves to be reviewed. Further complementary studies on plasma levels of doxycycline would be necessary to evaluate the efficacy of this drug in the prevention and treatment of lymphomas promoted by intracellular bacteria as *C. burnetii* and *M. tuberculosis*. Moreover, plasma levels of doxycycline and hydroxychloroquine should be systematically performed during treatment.

Finally, doxycycline, like hydroxychloroquine, is a safe and inexpensive drug with a minimal toxicity profile that deserves to be considered in the treatment of intracellular bacteria, which promote cancer, and in lymphoma induced by intracellular bacteria.
